# Comprehensive malnutritional index for predicting clinical outcomes in locally advanced rectal cancer receiving neoadjuvant chemoradiotherapy

**DOI:** 10.17305/bb.2024.11188

**Published:** 2024-10-17

**Authors:** Yu Xu, Peipei Shen, Jiahao Zhu, Danqi Qian, Ke Gu, Yong Mao, Shengjun Ji, Bo Yang, Yutian Zhao

**Affiliations:** 1 Department of Radiotherapy and Oncology, The Affiliated Hospital of Jiangnan University, Wuxi, China; 2 Wuxi Clinical Cancer Center, Wuxi, China; 3 Department of Oncology, The Affiliated Hospital of Jiangnan University, Wuxi, China; 4 Department of Radiotherapy and Oncology, The Affiliated Suzhou Hospital of Nanjing Medical University, Gusu School, Nanjing Medical University, Suzhou, China

**Keywords:** Comprehensive malnutritional index, locally advanced rectal cancer, malnutritional index, neoadjuvant chemoradiotherapy, prognosis

## Abstract

The objective of this investigation was to assess the prognostic significance of the comprehensive malnutritional index (CNI) in patients with locally advanced rectal cancer (LARC) who underwent neoadjuvant chemoradiotherapy (nCRT) followed by surgery. A total of 240 LARC patients were recruited. The CNI was calculated using principal components analysis based on hemoglobin (Hb), total lymphocyte count (TLC), albumin (ALB), body mass index (BMI), and usual body weight percentage (UBW%). The patients were then categorized into two groups based on the median CNI value. Cox regression and survival analyses were performed. The CNI-low (120 cases) and CNI-high (120 cases) groups were classified based on the median CNI value. The results indicated that the CNI demonstrated superior predictive ability for disease-free survival (DFS) and overall survival (OS) compared to other malnutritional indexes. LARC patients in the CNI-high group had significantly longer DFS and OS compared to those in the CNI-low group. Multivariate analysis revealed that the CNI was an independent prognostic factor for DFS (hazard ratio [HR] ═ 0.49; 95% confidence interval [CI], 0.29–0.83; *P* ═ 0.008) and OS (HR ═ 0.30; 95% CI, 0.16–0.58; *P* < 0.001). Additionally, the CNI-high group benefited from postoperative chemotherapy (DFS: *P* ═ 0.029, OS: *P* ═ 0.024), while the CNI-low group did not show such benefits (DFS: *P* ═ 0.448, OS: *P* ═ 0.468). These findings suggest that the CNI could serve as a valuable prognostic indicator for LARC patients who undergo nCRT followed by surgery. Preoperative nutrition optimization is important for LARC patients.

## Introduction

Despite advancements in routine screening and treatment, colorectal cancer remains the third leading cause of new cancer cases and cancer-related mortality [1]. This underscores the significant health burden posed by colorectal cancer; and highlights the necessity for ongoing prevention efforts and the development of effective treatment strategies to mitigate its impact. In China, rectal cancer accounts for approximately 50% of all colorectal cancer cases, with nearly 70% of patients presenting with locally advanced disease at diagnosis [2]. Traditional neoadjuvant therapy, which includes neoadjuvant chemoradiotherapy (nCRT) followed by surgery and subsequent adjuvant chemotherapy, as well as the recently proposed total neoadjuvant therapy where all chemotherapy cycles are administered preoperatively, have both shown efficacy in reducing local recurrence in cases of locally advanced rectal cancer (LARC). However, despite these treatments, approximately 30% of patients with LARC still suffer from distant metastasis [3, 4]. Therefore, there is an urgent need to identify more precise prognostic markers based on preoperative clinical parameters to better predict survival outcomes.

Recent studies have highlighted the relationship between pre-treatment malnutrition and cancer prognosis [5–8]. Patients with malnutrition often exhibit poorer treatment tolerance, prolonged hospital stays, increased medical costs, and reduced survival compared to those with adequate malnutritional status [9]. Thus, assessing the malnutritional risk of patients is crucial. It not only facilitates targeted malnutritional interventions but also significantly enhances clinical outcomes. Accurate evaluation ensures that malnutritional support is precisely tailored to meet the individual needs of each patient, thereby optimizing recovery and overall health. The European Society for Clinical Nutrition and Metabolism (ESPEN) recommends early screening of all cancer patients to identify potential malnutritional risks at the outset of treatment [10]. The use of preoperative malnutritional risk biomarkers has gained prominence in oncology for prognostication and survival prediction. For instance, a low prognostic nutritional index (PNI), calculated based on serum albumin (ALB) levels and total lymphocyte count (TLC), has been associated with poorer outcomes in breast cancer [11]. Wang et al. [12] reported that pretreatment PNI is a reliable predictor of both treatment response to nCRT and survival outcomes in patients with LARC. Patients with a high PNI demonstrated better tumor regression and longer survival. Similarly, a study by Lee et al. [13] found that dynamic PNI (dPNI) during nCRT also had predictive value for long-term outcomes, suggesting that mitigating malnutritional risk during concurrent chemoradiotherapy (CRT) could improve oncologic outcomes. The nutritional risk index (NRI), which is calculated using ALB and body weight, has also been identified as a malnutritional metric correlated with oncological outcomes in LARC [14, 15]. However, relying on a single parameter or a combination of two parameters may not sufficiently capture the complexity of malnutritional risk. Therefore, there is a pressing need to explore and develop a more comprehensive malnutritional index (CNI).

The comprehensive malnutrition index (CNI) has been used to evaluate the malnutritional risk in various types of cancers [16–18]. This index includes five nutrition-based indicators: body mass index (BMI), ALB, usual body weight percentage (UBW%), TLC, and hemoglobin (Hb). However, there is a lack of studies on the prognostic value of CNI in LARC patients undergoing nCRT. This study aims to explore whether CNI could serve as a reliable predictive marker for tumor response to neoadjuvant treatment as well as for survival outcomes. Additionally, we will compare the prognostic performance of CNI with PNI, NRI, and other nutrition indexes to identify the index that exhibits superior predictive capabilities.

## Materials and methods

### Patients

**Figure 1.**
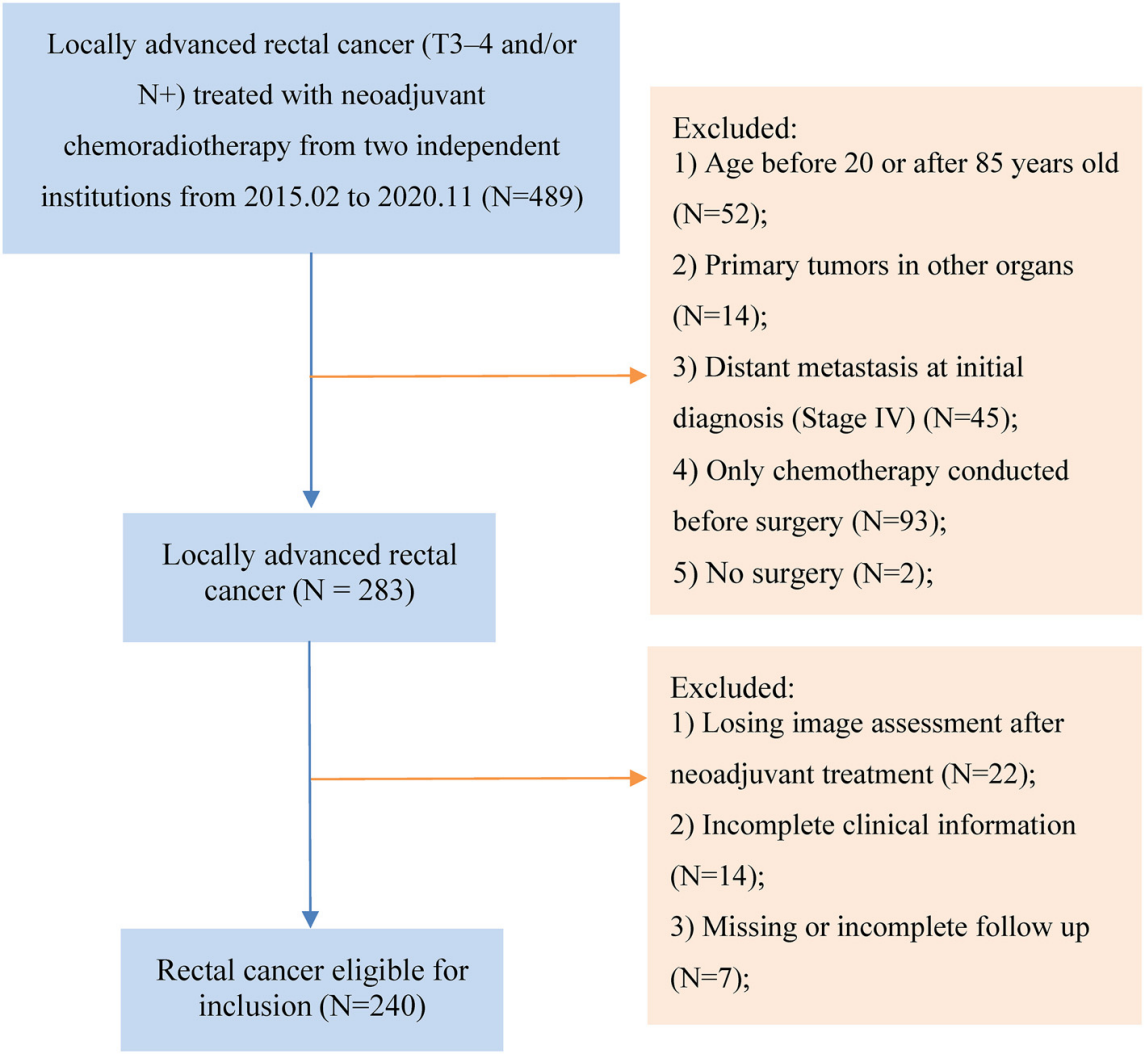
Flowchart of patient inclusion and exclusion criteria.

A retrospective analysis was conducted on a cohort of 240 patients who underwent nCRT followed by surgery for rectal cancer at the Affiliated Hospital of Jiangnan University and the Affiliated Suzhou Hospital of Nanjing Medical University between February 2015 and November 2020. Specific case selection criteria included: (1) histopathological confirmation of rectal cancer; (2) clinical stage II-III determined by magnetic resonance imaging (MRI), with tumor classification as cT3-4 and/or cN1-2; (3) age range of 20–85 years; (4) diagnosis of adenocarcinoma or mucinous adenocarcinoma; (5) absence of any other diagnosed cancer except rectal cancer; and (6) all patients underwent nCRT followed by radical surgery. The exclusion criteria included: (1) patients who received corticosteroids, ALB, statins, or malnutritional therapy during treatment; (2) missing data required for analysis; and (3) patients who underwent surgery at a different hospital. Figure 1 shows the flowchart of patient selection. The present study adhered to the Declaration of Helsinki and was approved by the Affiliated Hospital of Jiangnan University and the Affiliated Suzhou Hospital of Nanjing Medical University.

### Pathology assessment and survival outcome definition

The pathological stage of cancer patients was determined using the 8th edition staging system established by the American Joint Committee on Cancer as recommended by the National Comprehensive Cancer Network. Pathological complete response (pCR) was defined as the absence of any detectable residual tumor cells in both the primary tumor site and the resected lymph nodes within the surgical specimens. The study evaluated two survival outcomes: overall survival (OS) and disease-free survival (DFS). OS was calculated as the time from the operation to death or the last recorded visit, while DFS measured the duration from surgery for LARC to the first occurrence of disease recurrence or cancer-related death. Patients who completed the prescribed radiotherapy and chemotherapy were defined as treatment-completed patients.

### Treatment schedules and follow-up

All patients in the study received intensity-modulated radiotherapy (IMRT) using a Varian linear accelerator, delivering 6–10 MV X-rays over a period of approximately five weeks. A cumulative dose of 50 Gy was administered in 25 fractions during the radiotherapy sessions, while concurrently, oral capecitabine (825 mg/m^2^, twice daily) was given. Following the chemoradiation, some patients received two cycles of consolidation chemotherapy using the capecitabine and oxaliplatin (XELOX) regimen approximately 2–3 weeks later. Patients exhibiting high-risk factors, such as extramural vascular invasion, circumferential resection margin status, a greater number/proportion of involved lymph nodes, extra-nodal deposits, or poor tumor differentiation, were strongly recommended to receive two additional cycles of adjuvant chemotherapy with the XELOX regimen after surgery. Approximately 3–4 weeks after completing preoperative CRT, computed tomography scans of the chest, abdomen, and pelvis were performed to assess for possible metastasis. If no evidence of metastasis was detected, a total mesorectal excision was conducted around eight weeks after radiotherapy. Patients were subjected to regular follow-up assessments, at intervals of three months during the first two years, six months during years 3–5, and annually after five years. The final follow-up was conducted in May 2023.

### Malnutritional status assessment

Hb, TLC, and serum ALB measurements were collected from the hospitals’ clinical data repositories within one week prior to nCRT. BMI was calculated as weight (kg) divided by the square of height (meters). The UBW% was determined by calculating the ratio of the current body weight (CBW) to the usual body weight (UBW). UBW refers to the body weight recorded at initial admission. The specific formulas for calculating UBW%, PNI, and NRI can be found in Table S1.

### Construction of the CNI by principal component analysis (PCA)

In alignment with previous research, PCA was employed to calculate the CNI in MATLAB software (MATLAB R2020a) based on five nutritional parameters: BMI, UBW%, TLC, ALB, and Hb [16–18]. The goal was to derive a composite index that captures the maximum information regarding malnutrition status.

In performing PCA, the first step was to normalize the original data variables to ensure comparability on the same scale. Eigenvalues and their corresponding eigenvectors were then computed from the correlation coefficient matrix. Principal components were calculated, corresponding to the number of indicators. The eigenvalues represent the contribution of each principal component to the overall evaluation, stored in λ and arranged in descending order. Each eigenvector represents the correlation between a principal component and the original data. The larger the absolute value, the more representative the principal component is of the index. To simplify calculations, the sign of each eigenvector was adjusted so that the sum of its components is positive, yielding the final eigenvector. Based on these eigenvalues, the number of principal components to retain was determined, typically based on the cumulative explained variance. In this study, all principal components with eigenvalues greater than 1 and a cumulative contribution greater than 70% were selected. Using the selected eigenvectors, expressions for the principal components were formulated. Finally, the principal components were combined to establish the expression for the comprehensive index.

**Table 1:** Characteristics of 240 patients with locally advanced rectal cancer

**Characteristics**	***N* ═ 240**
*Age (years)*	
Mean ± SD	58.0 ± 10.0
≤ 60	131 (54.6%)
> 60	109 (45.4%)
*Gender*	
Male	144 (60.0%)
Female	96 (40.0%)
*Histology*	
Adenocarcinoma	231 (96.3%)
Mucinous adenocarcinoma	9 (3.8%)
*Differentiation*	
Well	23 (9.6%)
Moderate	195 (81.3%)
Poor	22 (9.2%)
*CEA pretreatment*	
≤ 5 µg/L	133 (55.4%)
> 5 µg/L	107 (44.6%)
*cT*	
cT3	171 (71.2%)
cT4	69 (28.8%)
*cN*	
cN0	77 (32.1%)
cN1	121 (50.4%)
cN2	42 (17.5%)
*cTNM*	
IIA	60 (25.0%)
IIB	15 (6.3%)
IIC	2 (0.8%)
IIIA	15 (6.3%)
IIIB	99 (41.3%)
IIIC	49 (20.4%)
*Treat response*	
pCR	45 (18.8%)
non-pCR	195 (81.3%)
*ypT*	
ypT0	45 (18.8%)
ypT1	17 (7.1%)
ypT2	31 (12.9%)
ypT3	132 (55.0%)
ypT4	15 (6.2%)
*ypN*	
ypN0	170 (70.8%)
ypN1	61 (25.4%)
ypN2	9 (3.8%)
*ypTNM*	
0	45 (18.8%)
I	34 (14.2%)
II	87 (36.3%)
III	74 (30.8%)
*Consolidation chemotherapy*	
Yes	29 (12.1%)
No	211 (87.9%)
*Postoperative chemotherapy*	
Yes	172 (71.7%)
No	68 (28.3%)
*Treatment completed*	
Yes	198 (82.5%)
No	42 (17.5%)
*Hemoglobin (g/L)*	
Mean ± SD	122.2 ± 16.1
Range	(84.3, 151.3)
*TLC (10^9^/L)*	
Mean ± SD	1.6 ± 0.4
Range	(0.5, 3.3)
*Albumin (g/L)*	
Mean ± SD	40.0 ± 5.6
Range	(26.6, 52.2)
*BMI (kg/m^2^)*	
Mean ± SD	22.9 ± 3.5
Range	(15.6, 34.1)
*UBW%*	
Mean ± SD	95.1 ± 4.5
Range	(81.1, 110.2)
*PNI*	
Mean ± SD	51.4 ± 5.8
Range	(34.2, 68.6)
*NRI*	
Mean ± SD	102.0 ± 8.0
Range	(81.5, 121.0)
*CNI*	
Mean ± SD	0.2 ± 1.2
Range	(-2.2, 2.3)

BMI: Body mass index; CEA: Carcinoembryonic antigen; CNI: Comprehensive malnutritional index; pCR: Pathologic complete response; PNI: Prognostic nutritional index; NRI: Nutrition risk index; SD: Standard deviation; TLC: Total lymphocyte count; UBW%: Usual body weight percentage.

The calculation expression for each principal component was obtained by using the characteristic contribution rate as the coefficient and the corresponding index as the independent variable. The comprehensive evaluation value was determined by taking the eigenvalue λ as the coefficient and the corresponding principal component as the independent variable.

### Ethical statement

The present study adhered to the Declaration of Helsinki and was approved by the Affiliated Hospital of Jiangnan University, and the Affiliated Suzhou Hospital of Nanjing Medical University. All participants provided informed consent.

### Statistical analysis

In the analysis of categorical variables, chi-squared tests or Fisher’s exact tests were used to assess the association between variables. For continuous variables, a *t*-test was employed to compare the means between groups. To compare the performance of the CNI with other malnutrition indicators, receiver operating characteristic (ROC) curves were constructed. The areas under the curves (AUCs) were calculated and compared to evaluate the discriminatory ability of the CNI compared to other indicators. The DFS and OS rates were calculated using the Kaplan–Meier method. Differences in survival rates between groups were assessed using log-rank tests. Univariate and multivariate logistic regression analyses were conducted to analyze the association between pCR and prognostic factors. For the analysis of DFS and OS, a Cox proportional hazards regression model was used. Both univariate and multivariate analyses were performed to examine the relationship between survival outcomes and prognostic factors. Factors with a *P* value less than 0.050 in the univariate analysis were included in the multivariable models. Statistical significance was defined as a two-sided *P* value of less than 0.050. All statistical analyses were performed using R, version 4.2.1.

**Figure 2.**
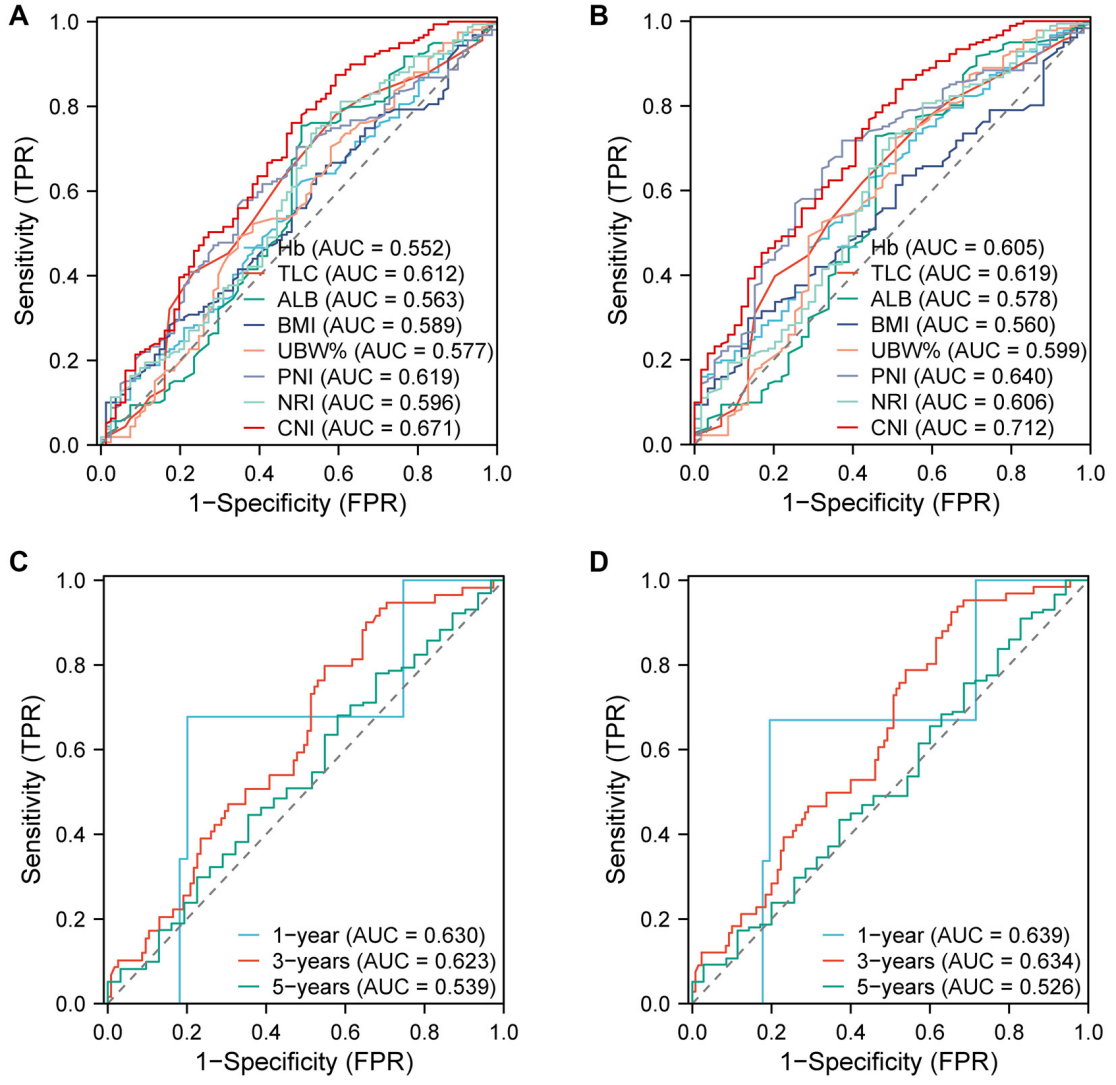
**AUC comparisons between CNI and other malnutrition indicators using ROC analysis.** (A) DFS; (B) OS. The prognostic value of CNI in time-dependent ROC analysis; (C) DFS; (D) OS. CNI: Comprehensive malnutritional index; DFS: Disease-free survival; OS: Overall survival; ROC: Receiver operating characteristic; AUC: Area under the curve.

**Table 2:** Characteristics of patients with locally advanced rectal cancer grouped by CNI

**Characteristic**	**CNI-low**	**CNI-high**	***P* value**
	***n* ═ 120**	***n* ═ 120**	
Age (years)			0.517
≤ 60	68 (56.6%)	63 (52.5%)	
> 60	52 (43.4%)	57 (47.5%)	
Gender			0.114
Male	78 (65.0%)	66 (55.0%)	
Female	42 (35.0%)	54 (45.0%)	
Differentiation			0.003
Well	5 (4.2%)	18 (15.0%)	
Moderate	99 (82.5%)	96 (80.0%)	
Poor	16 (13.3%)	6 (5.0%)	
CEA pretreatment			0.516
≤ 5 µg/L	64 (53.3%)	69 (57.5%)	
> 5 µg/L	56 (46.7%)	51 (42.5%)	
cT			0.377
cT3	82 (68.3%)	89 (74.2%)	
cT4	38 (31.7%)	31 (25.8%)	
cN			0.584
cN0	39 (32.5%)	38 (31.7%)	
cN1	63 (52.5%)	58 (48.3%)	
cN2	18 (15.0%)	24 (20.0%)	
cTNM			0.341
IIA	27 (22.5%)	33 (27.5%)	
IIB	10 (8.3%)	5 (4.2%)	
IIC	2 (1.7%)	0 (0%)	
IIIA	9 (7.5%)	6 (5.0%)	
IIIB	51 (42.5%)	48 (40.0%)	
IIIC	21 (17.5%)	28 (23.3%)	
Treatment response			0.072
pCR	17 (14.2%)	28 (23.3%)	
non-pCR	103 (85.8%)	92 (66.7%)	
ypT			0.222
ypT0	17 (14.2%)	28 (23.3%)	
ypT1	12 (10.0%)	5 (4.2%)	
ypT2	16 (13.3%)	15 (12.5%)	
ypT3	67 (55.8%)	65 (54.2%)	
ypT4	8 (6.7%)	7 (5.8%)	
ypN			0.572
ypN0	88 (73.3%)	82 (68.3%)	
ypN1	27 (22.5%)	34 (28.3%)	
ypN2	5 (4.2%)	4 (3.3%)	
Consolidation chemotherapy			0.166
Yes	11 (9.2%)	18 (15%)	
No	109 (90.8%)	102 (85%)	
Postoperative chemotherapy			0.197
Yes	91 (75.8%)	81 (67.5%)	
No	29 (24.2%)	39 (32.5%)	
PNI			0.022
Mean ± SD	51.1 ± 4.9	52.5 ± 4.5	
NRI			<0.001
Mean ± SD	100.3 ± 9.6	104.3 ± 8.5	
Hemoglobin (g/L)			0.006
Mean ± SD	119.3 ± 16.2	125.1 ± 16.2	
TLC (10^9^/L)			0.026
Mean ± SD	1.5 ± 0.4	1.7 ± 0.5	
Albumin (g/L)			0.020
Mean ± SD	39.1 ± 5.8	40.8 ± 5.3	
BMI (kg/m^2^)			0.031
Mean ± SD	22.9 ± 3.7	23.9 ± 3.4	
UBW%			0.018
Mean ± SD	94.5 ± 4.5	95.9 ± 4.6	

BMI: Body mass index; CEA: Carcinoembryonic antigen; CNI: Comprehensive malnutritional index; pCR: Pathologic complete response; PNI: Prognostic nutritional index; NRI: Nutrition risk index; SD: Standard deviation; TLC: Total lymphocyte count; UBW%: Usual body weight percentage.

## Results

### Patient characteristics

Table 1 displays the essential characteristics of the patients enrolled in this study. A total of 240 patients diagnosed with LARC were included, consisting of 144 male and 96 female patients. The average age at diagnosis was 58 years, with a range of 24–79 years. In this study, 29 patients (12.1%) received consolidation chemotherapy, and 172 patients (71.7%) were treated with postoperative chemotherapy. The median follow-up period was 35.2 months, ranging from 4 to 84 months. During the follow-up period, 81 individuals (33.8%) experienced recurrence, and 59 patients (24.6%) died from various causes. The mean PNI was 51.4, with values ranging from 34.2 to 68.6. The mean NRI was 102, with values ranging from 81.5 to 121.0.

### Construction of the CNI by PCA

In this study, the first three principal components were retained, accounting for 33.1%, 19.4%, and 17.8% of the original data’s malnutrition status, respectively. Together, these three components accounted for 70.3% of the total variance, indicating that they captured a substantial amount of information. The three principal components were computed using specific equations that assign weights to each of the five nutrition parameters. These weights were determined through PCA. The three principal components were computed using the following equations: C1 ═ 0.704 × A + 0.616 × B + 0.262 × C + 0.188 × D + 0.216 × E; C2 ═ 0.196 × A -- 0.265 × B + 0.688 × C + 0.315 × D + 0.715 × E; C3 ═ 0.211 × A + 0.268 × B + 0.801 × C + 0.106 × D -- 0.372 × E. The variables A, B, C, D, and E represent the normalized values of Hb, TLC, ALB, BMI, and UBW%, respectively.

By utilizing the statistical weight coefficients of these three principal components, the CNI was computed using a weighted sum. The equation for CNI was CNI ═ 0.331 × C1 + 0.194 × C2 + 0.178 × C3. This equation combines the information captured by each of the three principal components to generate a composite index that represents the individual’s clinical nutrition status.

The mean CNI was 0.20, with values ranging from --2.22 to 2.25, indicating that, on average, the study participants had a relatively moderate malnutrition status. The CNI provides a comprehensive assessment by considering multiple nutrition parameters and capturing their collective impact on overall malnutrition status.

### Prognostic comparison between CNI and other malnutritional indicators

ROC analyses were performed to compare the prognostic value of CNI and other malnutrition indicators, such as Hb, TLC, ALB, BMI, UBW%, PNI, and NRI. CNI was found to have the largest AUC and better prognostic ability than other malnutrition indicators for predicting DFS (Figure 2A) and OS (all *P* < 0.050) (Figure 2B). Figure 2C and 2D exhibits the time-dependent ROC curve. When comparing the AUC for predicting DFS and OS, the results of CNI vs other indicators—Hb, TLC, ALB, BMI, UBW%, PNI, and NRI—are as follows.

For DFS prediction, the standard error (SE) for CNI vs Hb is 0.051 with a 95% confidence interval (CI) of 0.012–0.219 (*P* ═ 0.019). For CNI vs TLC, the SE is 0.052 with a 95% CI of 0.008–0.151 (*P* ═ 0.043). For CNI vs ALB, the SE is 0.053 with a 95% CI of 0.003–0.213 (*P* ═ 0.042). For CNI vs BMI, the SE is 0.055 with a 95% CI of 0.013–0.232 (*P* ═ 0.027). For CNI vs UBW%, the SE is 0.053 with a 95% CI of 0.009–0.219 (*P* ═ 0.033). For CNI vs PNI, the SE is 0.073 with a 95% CI of 0.012–0.166 (*P* ═ 0.023). For CNI vs NRI, the SE is 0.051 with a 95% CI of 0.017–0.186 (*P* ═ 0.021).

For OS prediction, the SE for CNI vs Hb is 0.053 with a 95% CI of 0.001–0.212 (*P* ═ 0.047). For CNI vs TLC, the SE is 0.054 with a 95% CI of 0.004–0.209 (*P* ═ 0.045). For CNI vs ALB, the SE is 0.056 with a 95% CI of 0.025–0.249 (*P* ═ 0.016). For CNI vs BMI, the SE is 0.058 with a 95% CI of 0.048–0.274 (*P* ═ 0.005). For CNI vs UBW%, the SE is 0.062 with a 95% CI of 0.001–0.246 (*P* ═ 0.048). For CNI vs PNI, the SE is 0.056 with a 95% CI of 0.009–0.253 (*P* ═ 0.045). For CNI vs NRI, the SE is 0.055 with a 95% CI of 0.007–0.225 (*P* ═ 0.036).

### Characteristics grouped by CNI

**Figure 3.**
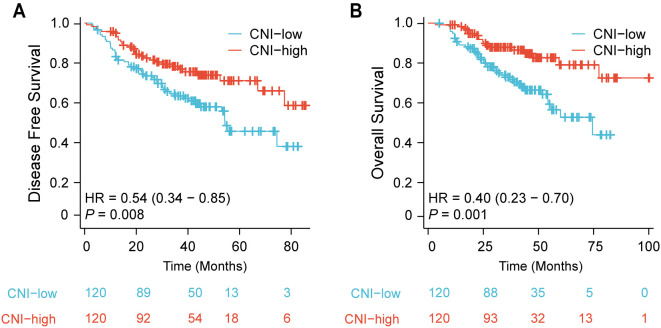
**Differences in DFS (A) and OS (B) between CNI-low group and CNI-high LARC patients treated with neoadjuvant chemoradiotherapy.** CNI: Comprehensive malnutritional index; DFS: Disease-free survival; OS: Overall survival; LARC: Locally advanced rectal cancer.

Based on the median value of CNI, patients were divided into low (CNI < 0.108) and high (CNI > 0.108) groups. Differences in clinicopathological characteristics between the two groups are displayed in Table 2. Differentiation, PNI, NRI, Hb, TLC, ALB, BMI, and UBW% showed significant differences (*P* < 0.050). LARC patients with high CNI were found to have better DFS (*P* ═ 0.008) and OS (*P* ═ 0.001) compared to low CNI individuals. The Kaplan–Meier curves of DFS and OS are shown in Figure 3A and 3B.

**Figure 4.**
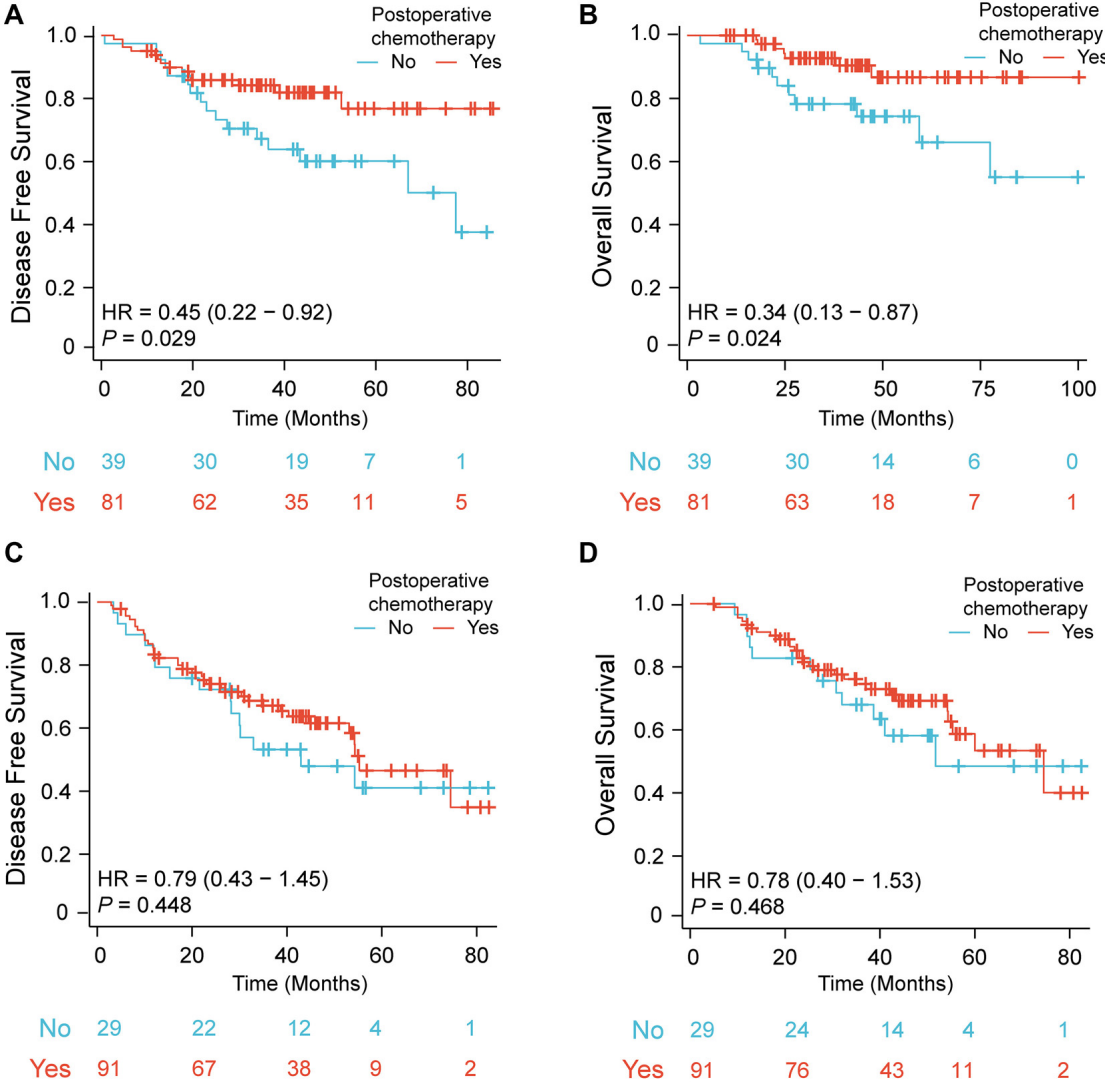
Differences in DFS (A) and OS (B) between LARC patients treated with postoperative chemotherapy and those who did not receive postoperative adjuvant chemotherapy in the CNI-high subgroup. Differences in DFS (C) and OS (D) between LARC patients treated with postoperative chemotherapy and those who did not receive postoperative adjuvant chemotherapy in the CNI-low subgroup. CNI: Comprehensive malnutritional index; DFS: Disease-free survival; OS: Overall survival; LARC: Locally advanced rectal cancer.

Further analysis demonstrated that LARC patients who received postoperative chemotherapy had better DFS (*P* ═ 0.029) and OS (*P* ═ 0.024) compared with those who did not receive adjuvant chemotherapy in the CNI-high subgroup (Figure 4A and 4B). No difference was observed in the CNI-low subgroup, regardless of whether these patients received chemotherapy after surgery (DFS: *P* ═ 0.448; OS: *P* ═ 0.468) (Figure 4C and 4D).

### Logistic and Cox outcomes for independent prognostic factors

Univariate and multivariate logistic analyses for pCR showed that clinical lymph node metastasis state (cN) was an independent predictor (odds ratio [OR] ═ 2.26; 95% CI, 1.03–5.77; *P* ═ 0.032) (Table S2). Univariate Cox analyses for DFS (Table 3) and OS (Table 4) showed that treatment response, ypN, and CNI were important predictors for DFS. Meanwhile, cN, treatment response, ypN, and CNI were important predictors for OS. Multivariate Cox analyses demonstrated that treatment response (hazard ratio [HR] ═ 2.56; 95% CI, 1.65–4.36; *P* ═ 0.002), ypN (HR ═ 1.70; 95% CI, 1.14–3.07; *P* ═ 0.012), and CNI (HR ═ 0.49; 95% CI, 0.29–0.83; *P* ═ 0.008) served as independent predictors for DFS. cN (HR ═ 1.15; 95% CI, 1.02–2.11; *P* ═ 0.035), treatment response (HR ═ 1.38; 95% CI, 1.03–2.46; *P* ═ 0.031), ypN (HR ═ 2.98; 95% CI, 1.54–5.79; *P* ═ 0.001), and CNI (HR ═ 0.30; 95% CI, 0.16–0.58; *P* < 0.001) were independent predictors for OS.

**Table 3:** Univariate and multivariate Cox analysis for DFS in patients with locally advanced rectal cancer

**Characteristic**	**Univariable (DFS)**		**Multivariable (DFS)**	
	**HR (95% CI)**	***P***	**HR (95% CI)**	***P***
*Age*				
≤ 60	ref			
> 60	1.16 (0.74–1.80)	0.516		
*Gender*				
Male	ref			
Female	1.14 (0.72–1.81)	0.583		
*Differentiation*				
Well	ref			
Moderate	1.07 (0.49–2.34)	0.862		
Poor	1.47 (0.55–3.94)	0.447		
*CEA pretreatment*				
≤ 5 µg/L	ref			
> 5 µg/L	1.31 (0.85–2.03)	0.219		
*cT*				
cT3	ref			
cT4	0.94 (0.38–2.33)	0.897		
*cN*				
cN0	ref			
cN+	2.44 (0.44–1.24)	0.414		
*Treatment response*				
pCR	ref		ref	
non-pCR	1.55 (1.82–2.92)	0.006	2.56 (1.65–4.36)	0.002
*ypT*				
ypT0	ref			
ypT1-2	1.66 (0.61–4.50)	0.320		
ypT3-4	1.57 (0.69–3.56)	0.280		
*ypN*				
ypN0	ref		ref	
ypN+	1.62 (1.01–2.63)	0.049	1.70 (1.14–3.07)	0.012
*Consolidation chemotherapy*				
Yes	ref	0.788		
No	1.05 (0.54–1.88)			
*Postoperative chemotherapy*				
Yes	ref			
No	1.25 (0.84–2.74)	0.146		
*CNI*				
Low	ref		ref	
High	0.54 (0.34–0.85)	0.008	0.49 (0.29–0.83)	0.008

CEA: Carcinoembryonic antigen; CNI: Comprehensive malnutritional index; pCR: Pathologic complete response; ref: Reference; DFS: Disease-free survival; HR: Hazard ratio; CI: Confidence interval.

**Table 4:** Univariate and multivariate Cox analysis for OS in patients with locally advanced rectal cancer

**Characteristic**	**Univariable (OS)**		**Multivariable (OS)**	
	**HR (95% CI)**	***P***	**HR (95% CI)**	***P***
*Age*				
≤ 60	ref			
> 60	1.29 (0.77–2.16)	0.332		
*Gender*				
Male	ref			
Female	1.36 (0.78–2.38)	0.283		
*Differentiation*				
Well	ref			
Moderate	0.88 (0.38–2.07)	0.772		
Poor	1.63 (0.56–4.70)	0.367		
*CEA pretreatment*				
≤ 5 µg/L	ref			
> 5 µg/L	1.23 (0.74–2.05)	0.432		
*cT*				
cT3	ref			
cT4	1.14 (0.36–3.65)	0.824		
*cN*				
cN0	ref		ref	
cN+	2.17 (1.19–4.01)	0.012	1.15 (1.05–2.11)	0.035
*Treatment response*				
pCR	ref		ref	
non-pCR	1.29 (1.01–1.81)	0.042	1.38 (1.03–2.46)	0.031
*ypT*				
ypT0	ref			
ypT1-2	2.51 (0.76–8.25)	0.130		
ypT3-4	1.60 (0.54–4.76)	0.399		
*ypN*				
ypN0	ref		ref	
ypN+	2.35 (1.37–4.01)	0.002	2.98 (1.54–5.79)	0.001
*Consolidation chemotherapy*				
Yes	ref	0.675		
No	1.16 (0.64–2.03)			
*Postoperative chemotherapy*				
Yes	ref			
No	1.49 (0.93–3.51)	0.119		
*CNI*				
Low	ref		ref	
High	0.40 (0.23–0.70)	0.001	0.30 (0.16–0.58)	<0.001

CEA: Carcinoembryonic antigen; CNI: Comprehensive malnutritional index; pCR: Pathologic complete response; ref: Reference; OS: Overall survival; HR: Hazard ratio; CI: Confidence interval.

## Discussion

In this study, we first investigated the association between CNI and prognosis in LARC patients treated with nCRT followed by surgery. The prediction efficiency of CNI was compared with other malnutrition indicators, such as Hb, TLC, ALB, BMI, UBW%, PNI, and NRI. We observed that CNI had better predictive ability for DFS and OS than these common malnutrition indicators and could serve as an independent prognostic factor for DFS and OS. Therefore, CNI may be a promising malnutrition marker to distinguish LARC patients with poorer long-term prognoses. Additionally, subgroup analysis demonstrated that LARC patients with high CNI could benefit from postoperative chemotherapy, while those in the low CNI group did not. Malnutrition, rather than malignancy itself, accounts for an estimated 10%–20% of mortality in cancer patients [10]. nCRT prior to surgery is the standard treatment for LARC, yet gastrointestinal toxicities, including anorexia, nausea, and vomiting, are major adverse events during therapy. These side effects can be troublesome for patients and may impact their overall well-being and nutritional status. Early identification of malnourished individuals or those at risk of malnutrition is crucial for the timely implementation of nutritional interventions, ultimately leading to improved prognosis and decreased medical costs. The prognostic predictive power of several malnutrition indexes in cancer has been explored. BMI is widely utilized for evaluating an individual’s nutritional status. Low BMI is associated with malnutrition, sarcopenia, and metabolic disorders [19]. A low preoperative BMI has served as an unfavorable prognostic indicator for patients with gastric cancer [20]. Circulating Hb not only reflects tumor oxygenation levels but also indicates a lack of iron intake and chronic protein deficiency. Poor treatment response and survival outcomes were observed in LARC patients with low Hb undergoing nCRT [21]. Both lymphocytes and ALB could reflect nutritional status and systemic immune responses. These two malnutrition indicators were reported to have a significant association with LARC prognosis, though only ALB replenishment could be efficiently delivered [21]. UBW% mainly reflects changes in body weight after diagnosis and before treatment. Individual nutrition parameters, as well as the PNI and NRI derived from two nutrition indicators, fail to offer a comprehensive assessment of malnutrition status. CNI, composed of five parameters (BMI, UBW%, Hb, TLC, and ALB), can more comprehensively reflect the body’s malnutrition status. Recent studies demonstrated that patients with low CNI had poor survival outcomes in nasopharyngeal carcinoma (NPC) [16], hepatocellular carcinoma (HCC) [17], and esophageal squamous cell carcinoma (ESCC) [18]. The study design of Feng et al. was similar to ours. A total of 233 ESCC patients receiving neoadjuvant immunotherapy combined with chemotherapy (nICT) were included, and the association between CNI and treatment response and survival outcomes was investigated. They found that the high CNI group had a higher pCR rate, longer OS and DFS, and fewer postoperative complications compared with the low CNI group [18]. A more advanced cancer stage was also found in CNI-low groups. Our study also exhibited better OS and DFS rates in CNI-high LARC patients, but no difference in pCR rates existed between the two groups. We suggest that different methods of threshold determination may have contributed to this difference. The optimal cut-off was defined using a cut-off finder in the ESCC study, while in our study, threshold determination was based on the median value. Additionally, the prognostic value of CNI for survival prediction was observed to be better than that of NRI and PNI in these studies, which is in line with the findings of the present study. Hence, the CNI serves as a promising indicator for evaluating malnutrition status and predicting the prognosis of cancer patients. It is imperative that the assessment of malnutrition status extends beyond the mere collection of BMI and ALB levels. Additional data points, such as UBW%, TLC, and Hb levels, should be incorporated to provide a more comprehensive evaluation. The calculation of CNI offers several advantages compared to other malnutrition indices like PNI and NRI. CNI provides a comprehensive assessment of malnutrition status by considering multiple nutrition parameters through PCA. This comprehensive approach captures the collective impact of these parameters, providing a more holistic view of an individual’s malnutrition health. In contrast, PNI and NRI typically rely on simpler formulas based on fewer parameters, which may result in a less comprehensive assessment. However, the complexity of CNI’s calculation can be a disadvantage, making it more challenging to interpret and apply in practical settings. Healthcare professionals may find it difficult to use CNI due to its intricacy. Additionally, CNI lacks the standardization and widespread validation seen in more established indices like PNI and NRI, which have been widely used and validated in various clinical settings and populations. On the other hand, PNI and NRI offer advantages in terms of simplicity and established use. They are relatively easy to calculate and interpret, making them more accessible for healthcare professionals. However, their simplicity may limit the scope of the assessment and potentially result in information loss. Ultimately, the choice of index depends on the specific clinical context and the need for a comprehensive vs simplified assessment of malnutrition status. While CNI provides a comprehensive assessment, its complexity and lack of standardization may pose challenges, whereas PNI and NRI offer simplicity and established use but may provide a more limited assessment.

The administration of nCRT followed by total mesorectal excision significantly decreased the locoregional recurrence to about 10%, yet distant recurrence remains high, which serves as the primary factor contributing to mortality in LARC [22]. Most LARC patients received the standard treatment strategy, and about 12% received two cycles of consolidation chemotherapy with the XELOX regimen between the period of nCRT and surgery. Consistent with our previous findings, the administration of consolidation chemotherapy before surgery did not have a significant impact on survival outcomes for LARC patients [23]. The decision of whether to treat patients with adjuvant chemotherapy after surgery depends on individual clinicopathological risk factors. Several recent studies demonstrated the feasibility and effectiveness of the total neoadjuvant therapy strategy, which involves administering both chemotherapy and radiation therapy before surgical intervention in LARC [24, 25]. Our previous study, along with research by Lo et al., demonstrated the advantages of achieving pCR with tolerable toxicities when applying this therapeutic strategy in the treatment of LARC [23, 24]. When patients exhibit good tolerance to chemotherapy, administering additional cycles of induction chemotherapy prior to concurrent CRT may positively impact their nutritional status. This can create a beneficial cycle of improved performance status, enhanced tolerance, and better treatment response. Additionally, this current study found that patients who received postoperative chemotherapy had better DFS and OS in the high CNI group but not in the low CNI group, though postoperative chemotherapy was not an independent factor for DFS and OS across the entire population. This suggests that CNI should be taken into consideration as an important factor before adjuvant therapy, and timely nutritional supplementation during the treatment period is necessary for CNI-low LARC patients. The impact of poor malnutrition status on cancer prognosis is multifaceted and significant. In cancer patients, malnutrition is closely linked to a weakened immune system, which can hinder the body’s ability to fight infections and respond effectively to cancer treatments. Additionally, malnourished patients often experience longer recovery periods and reduced resilience to the adverse effects of treatment, such as fatigue and nausea. Research has shown that inadequate nutrition can account for 10%–20% of cancer-related mortality, highlighting its critical role in patient outcomes [26]. Furthermore, cancer treatments, including chemotherapy and radiation, can worsen malnutrition status by causing side effects like loss of appetite, taste changes, and gastrointestinal issues, which further complicate the patient’s ability to maintain sufficient nutritional intake. In our study, we observed that a higher percentage of patients in the CNI-high group, identified as having poor nutritional status, struggled to complete their prescribed chemotherapy or CRT regimens compared to those in the CNI-low group. This suggests that patients with LARC and poor nutritional status exhibit lower treatment tolerance, leading to interruptions or modifications in their treatment plans, which can adversely affect their overall prognosis and chances of recovery. These findings underscore the importance of early nutritional assessment and intervention as part of comprehensive cancer care. By addressing nutritional deficiencies and supporting optimal dietary intake, healthcare providers can potentially improve treatment adherence, enhance patient well-being, and ultimately contribute to better clinical outcomes for cancer patients.

For patients with poor nutritional status, there currently lacks a standard treatment approach. Immunotherapy is gradually being applied in colorectal cancer due to its better therapeutic efficacy and lower side effects [27, 28]. A novel treatment strategy proposed by Lin et al. may be a better choice for LARC patients with poor nutrition due to the shorter radiation therapy course and milder toxic side effects [29]. Patients were administered 5 × 5 Gy of short-course radiotherapy followed by two 21-day cycles of CAPOX (a chemotherapy regimen) plus camrelizumab, with radical surgery conducted one week thereafter. The pCR rate reached 48.1%, including 46.2% for proficient mismatch repair tumors and 100% for deficient mismatch repair tumors, without severe adverse events, suggesting this treatment strategy could be prioritized for those with poor nutritional status. Currently, the efficacy of postoperative adjuvant immunotherapy is unknown, but adjuvant chemotherapy is still recommended for patients with a high risk of recurrence. Additionally, regular monitoring of nutritional status and implementing early interventions are crucial components in the overall management of patients with LARC. Proper nutritional support can improve treatment tolerance, enhance recovery, and potentially lead to better overall outcomes. Therefore, a multidisciplinary approach that includes dietitians and nutrition specialists is essential to identify and address nutritional deficiencies early in the treatment process. Our study is the first to investigate the association between CNI prior to treatment and tumor regression and survival prognosis in LARC patients who underwent nCRT. However, it is important to acknowledge several limitations within this study. Firstly, the retrospective design introduces potential selection bias and information bias. Secondly, while we excluded patients with hematological disorders or those undergoing immunomodulatory treatment, it is worth noting that other conditions may still impact blood-based biomarkers, such as diabetes. However, the diagnosis of diabetes in tumor patients cannot be simply considered a direct influencing factor. Clinically, poor blood glucose control affects all bodily systems, subsequently influencing hematological indicators. Given that all tumor patients diagnosed with diabetes in this study received hypoglycemic treatment and routine blood glucose monitoring, we believe this factor has minimal impact on the study’s results. Thirdly, the limited sample size and lack of external validation may restrict the generalizability of our findings. Fourthly, we did not collect data on treatment complications. Lastly, although we used the weight at admission (pre-treatment) as the baseline for UBW to minimize systematic error, deviations may exist due to potential weight loss before treatment initiation. Other potential influencing factors include psychological conditions, such as depression or anxiety after tumor diagnosis, which can affect patients’ appetites. However, these influences can vary with changes in the surrounding environment, such as support from family and friends. Despite the availability of numerous survey scales, the results are often too subjective to objectively assess patients’ true psychological states. In summary, the factors affecting nutritional indicators in the blood are multifaceted and highly complex. Clinically, it is established that the side effects of radiotherapy and chemotherapy, such as nausea and vomiting, significantly impact patients’ hematological indicators. Therefore, this study primarily focuses on analyzing nutritional indicators before treatment to predict tumor prognosis.

## Conclusion

In summary, pre-treatment CNI shows promise as a suitable indicator reflecting the overall nutritional status of patients with LARC. CNI demonstrates significant predictive value for both DFS and OS in LARC patients who undergo nCRT followed by surgery. It is worth noting that LARC patients with a low CNI may not benefit from postoperative chemotherapy and require timely nutritional interventions. However, further studies are necessary to validate and reinforce these findings.

## Supplemental data

**Table S1:** Nutritional markers of interest

**Nutritional marker**	**Parameters**	**Formula**
Usual body weight percentage (UBW%)	Current body weight; Usual body weight	Current body weight (kg)/Usual body weight (kg)
Prognostic nutritional index (PNI)	Serum ALB; Lymphocyte count	ALB (g/L) + 0.005 × TLC (count/µL)
Nutritional risk index (NRI)	Serum ALB; Usual body weight	(1.519 × ALB, g/L) + (41.7 × present/usual body weight)

TLC: Total lymphocyte count; ALB: Albumin.

**Table S2:** Univariate and multivariate logistic analysis for pCR in locally advanced rectal cancer

**Characteristic**	**Univariable (pCR)**		**Multivariable (pCR)**	
	**HR > (95% CI)**	***P***	**HR (95% CI)**	***P***
*Age*				
≤ 60	ref			
> 60	0.85 (0.44–1.63)	0.633		
*Gender*				
Male	ref			
Female	1.40 (0.72–2.69)	0.312		
*Differentiation*				
Well	ref			
Moderate	0.90 (0.33–2.86)	0.814		
Poor	0.17 (0.01–1.19)	0.122		
*CEA pretreatment*				
≤ 5 µg/L	ref			
> 5 µg/L	0.71 (0.36–1.37)	0.310		
*cT*				
cT3	ref			
cT4	0.61 (0.20–2.30)	0.421		
*cN*				
cN0	ref		ref	
cN+	1.92 (1.01–4.11)	0.046	2.26 (1.03–5.77)	0.032
*CNI*				
Low	ref			
High	1.29 (0.51–2.23)	0.302		

CEA: Carcinoembryonic antigen; CNI: Comprehensive nutritional index; N: Lymph node metastasis stage; pCR: Pathologic complete response; SD: Standard deviation; T: Tumor stage; TNM: Tumor node metastasis stage; pCR: Pathological complete response; HR: Hazard ratio; CI: Confidence interval.

## Data Availability

The data that support the findings of this study are not openly available due to reasons of sensitivity and are available from the corresponding author upon reasonable request.
